# Gut microbiome and its metabolites in liver cirrhosis: mechanisms and clinical implications

**DOI:** 10.3389/fcimb.2025.1717696

**Published:** 2025-11-06

**Authors:** Luyuan Chang, Yang Liu, Haipeng Li, Jiaqi Yan, Wenzong Wu, Nuo Chen, Chunyu Ma, Xinyi Zhao, Juan Chen, Jing Zhang

**Affiliations:** 1 The First Department of Clinical Medicine, Bengbu Medical University, Bengbu, Anhui, China; 2 School of Mental Health, Bengbu Medical University, Bengbu, Anhui, China

**Keywords:** cirrhosis, gut liver axis, intestinal dysbiosis, bile acid signaling, fecal microbiota transplantation

## Abstract

Cirrhosis remains a significant global health burden, causing approximately 1.4–1.5 million deaths each year and contributing to nearly 46 million disability-adjusted life years (DALYs) worldwide. Increasing evidence identifies the gut–liver axis as a central driver of disease progression, wherein intestinal dysbiosis, barrier disruption, and microbe-derived metabolites collectively exacerbate inflammation, fibrogenesis, and related complications. Across more than 40 recent studies, gut microbial α-diversity declined by 30–60%, and over 80% reported a marked depletion of short-chain fatty acid (SCFA)–producing taxa, particularly Lachnospiraceae and Ruminococcaceae. Meta-analyses indicate that fecal butyrate levels decrease by 40–70%, accompanied by a two- to fourfold increase in endotoxin concentrations. Bile acid profiling demonstrates an approximately 50% reduction in secondary bile acids and significant suppression of FXR/TGR5 signaling, whereas tryptophan metabolism shifts toward the kynurenine pathway, weakening epithelial defense and exacerbating portal hypertension. Clinically, dysbiosis and microbial translocation are associated with higher MELD scores, and patients in the lowest quartile of microbial diversity have a threefold increased risk of hepatic encephalopathy or spontaneous bacterial peritonitis. Microbiome-targeted interventions—including lactulose, rifaximin, probiotics or synbiotics, fecal microbiota transplantation, and bile acid modulators—restore community balance in 70–85% of clinical trials, although efficacy and safety vary by etiology and baseline microbiota composition. Integrated microbiome–metabolome models achieve areas under the curve (AUCs) of 0.82–0.90 for noninvasive classification and early detection of cirrhosis. Collectively, these findings underscore reproducible, quantitative microbiome–metabolite alterations and outline a roadmap for microbiome-informed precision care that connects mechanistic insight with clinical application, emphasizing the need for longitudinal and multi-ethnic validation.

## 1 Introduction

Liver cirrhosis remains a major global health challenge. Contemporary estimates indicate about 1.43 to 1.47 million deaths each year and about 46 million disability adjusted life years, abbreviated as DALYs, attributable to cirrhosis and other chronic liver diseases. Incident cases exceeded 58 million in 2021 (Tham et al., 2025). Although age standardized mortality and DALY rates have declined modestly since 2010, the absolute burden continues to rise as populations age and metabolic risks expand (Devarbhavi et al., 2023; Danpanichkul et al., 2025). Marked geographic heterogeneity reflects differences in viral hepatitis control, alcohol use, obesity prevalence, and health system capacity. Etiologic patterns are shifting. As HBV and HCV burdens fall in many regions, alcohol associated liver disease and metabolic dysfunction associated steatotic liver disease, MASLD, formerly NAFLD, increasingly drive incident cirrhosis and decompensation (Rinella et al., 2023). Therapeutic options lag behind clinical need. Portal hypertension, the hemodynamic hallmark of decompensation, lacks effective pharmacologic therapies; management therefore remains largely supportive (Elfeki et al., 2023; Porada and Bułdak, 2025). Liver transplantation, constrained by limited access and high wait list mortality, remains the only curative option for end stage disease (Choudhury et al., 2024; Vitale et al., 2024).

Against this backdrop, the gut liver axis has emerged as a central pathophysiological framework linking intestinal dysbiosis, barrier dysfunction, microbial translocation, and liver injury (Hsu and Schnabl, 2023; Rodrigues et al., 2024). Recent advances indicate that alterations in microbial communities and metabolites, including short chain fatty acids, altered bile acid pools that perturb FXR and TGR5 signaling, and tryptophan derived indoles, amplify endotoxemia, hepatic inflammation, and fibrogenesis (Fang et al., 2022; Gao et al., 2024; Münte and Hartmann, 2025). These changes exacerbate portal hypertension and complications such as hepatic encephalopathy (Shah et al., 2024; Kronsten et al., 2025). Supported by multi omics and mechanistic studies, these insights have catalyzed translational efforts to restore eubiosis, strengthen the intestinal barrier, and attenuate microbe driven inflammation in cirrhosis.

**Figure 1:**
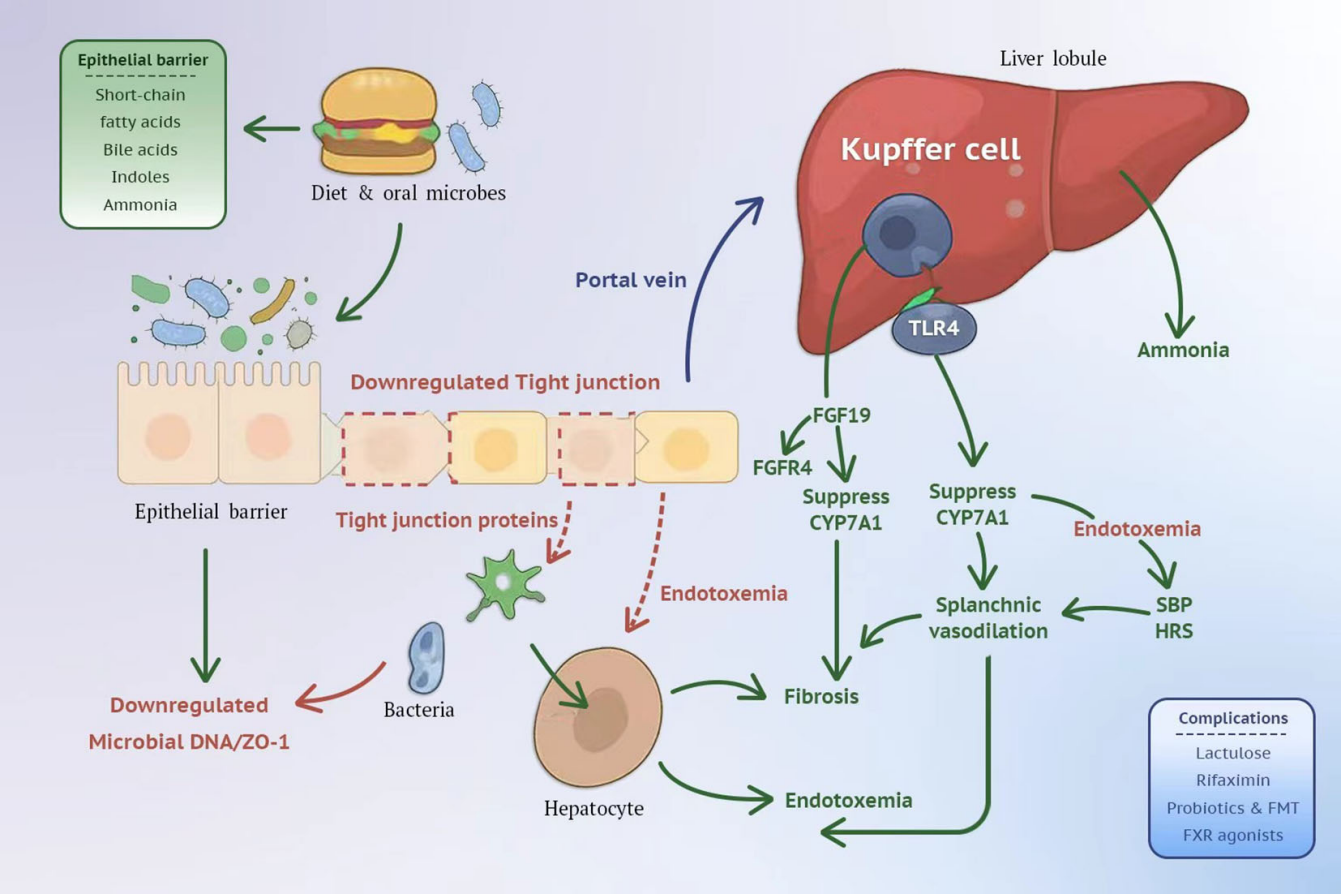
Gut–liver axis in cirrhosis. Dysbiosis weakens the epithelial barrier, allowing bacterial DNA/LPS to translocate via the portal vein. Hepatic TLR4 activation and FXR–FGF19/FGFR4–CYP7A1 dysregulation drive inflammation, fibrosis, endotoxemia, and portal-hypertensive complications (SBP, HRS); ammonia exacerbates HE. Key interventions target these nodes: lactulose, rifaximin, probiotics/FMT, FXR agonists.

Accordingly, this review synthesizes advances from 2022 to 2025 on the roles of the gut microbiome and its metabolites in liver cirrhosis (Xu et al., 2025b). A mechanistic model links dysbiosis to portal hypertension via barrier failure, microbial translocation, and hepatic immune activation (**Figure 1**). We integrate mechanistic evidence along the gut liver axis, appraise clinical data linking dysbiosis and metabolites to disease severity and complications (Zhu et al., 2024; Lee et al., 2025), and evaluate the therapeutic landscape, from established gut directed strategies such as lactulose and rifaximin to emerging approaches such as fecal microbiota transplantation and bile acid pathway modulators (Hoilat et al., 2022; Adorini and Trauner, 2023; Samanta and Sen Sarma, 2024; Bajaj et al., 2025). We highlight opportunities and challenges for microbiome informed precision care in cirrhosis.

## 2 Gut microbiota dysbiosis and barrier dysfunction in cirrhosis

### 2.1 Altered microbial composition

Multiple recent studies report that cirrhosis is consistently accompanied by marked gut dysbiosis (Lee et al., 2025**).** Alpha diversity of the gut microbiota is significantly lower in patients with cirrhosis than in healthy controls (Liu et al., 2025). At the taxonomic level, beneficial Firmicutes decline, especially short chain fatty acid producing families such as Lachnospiraceae and Ruminococcaceae, along with Bifidobacteria, whereas potentially pathogenic Bacteroidetes and Proteobacteria increase (Jung et al., 2022). For example, several studies describe reduced Clostridium IV, Coprococcus, and other commensals, together with relative overgrowth of Enterobacteriaceae, Veillonella, Streptococcus, and Prevotellaceae in cirrhosis (Wu et al., 2023). This dysbiotic profile worsens as cirrhosis progresses (Xu et al., 2025c). Decompensated disease shows the most pronounced shifts, with near loss of many anaerobic short chain fatty acid producers. Jiang and colleagues observed decreased Firmicutes and increased Bacteroidetes at the phylum level in patients with cirrhosis (Yu et al., 2025). These compositional shifts parallel metabolic changes. Loss of short chain fatty acid producers correlates with lower butyrate levels and weakened intestinal barrier function (Pant et al., 2023; Münte and Hartmann, 2025). Most evidence derives from human cross-sectional and prospective cohort studies that document reduced α-diversity and characteristic taxonomic shifts. Randomized trials primarily evaluate microbiome-targeted interventions rather than baseline composition. Mechanistic and animal models provide convergent support for causality through epithelial-barrier dysfunction and microbial translocation. In summary, cirrhosis associated dysbiosis shows a characteristic profile that tracks with disease severity (**Table 1**).

**Table 1:** Dysbiosis signatures repeatedly reported in liver cirrhosis.

Taxon/Feature	Direction in cirrhosis	Sample site	Associated clinical features	Notes
Ruminococcaceae	decrease	Stool	Lower SCFAs; linked to HE and fibrosis severity	SCFA-producing; depletion common
Lachnospiraceae	decrease	Stool	Barrier integrity loss; endotoxemia	SCFA-producing; butyrate deficit
Bifidobacterium	decrease	Stool	Worse cognitive scores in HE	Prebiotic-responsive genus
Akkermansia muciniphila	decrease	Stool	Thinner mucus layer; permeability ↑	Mucin degrader; often reduced
Enterobacteriaceae (Escherichia/Shigella)	increase	Stool, blood	Endotoxemia; inflammation	LPS source; pathobionts
Streptococcus (oralization)	increase	Stool, duodenum	Associated with decompensation risk	Translocation from oral cavity
Veillonella (oralization)	increase	Stool, duodenum	Portal hypertension, HE	Lactate utilizer; oralization marker
Klebsiella pneumoniae (high-alcohol-producing)	increase	Stool	Steatohepatitis features; oxidative stress	Endogenous ethanol production
Proteobacteria (phylum)	increase	Stool	Dysbiosis index ↑	Broad LPS-rich expansion

### 2.2 Barrier dysfunction and translocation

Cirrhosis impairs the intestinal epithelial barrier and creates a leaky gut that permits microbial translocation (Costa et al., 2025a; Haedge et al., 2025). Structural alterations develop under portal hypertension and inflammation, including reduced mucus production and damage to tight junctions (Hodgkinson et al., 2023; Costa et al., 2025a). Dysbiosis worsens barrier injury by reducing production of short chain fatty acids and by increasing mucolytic and pro inflammatory bacteria; butyrate normally supports tight junction assembly (Peng et al., 2009). Zhu and colleagues reported that lower intraluminal bile acids due to cholestasis together with dysbiosis reduce barrier integrity (Zhu et al., 2023). In cirrhosis, endotoxemia defined as elevated plasma lipopolysaccharide is common and correlates with disease severity and with minimal hepatic encephalopathy (Efremova et al., 2024; Ntuli and Shawcross, 2024). Clinical studies show that patients with cirrhosis often have increased bacterial DNA or endotoxin in blood and in ascitic fluid, even in the absence of overt infection, which indicates ongoing microbial leakage (Francés et al., 2008; González-Navajas et al., 2008). This translocation triggers hepatic immune activation (Haedge et al., 2025). Pathogen associated molecular patterns such as lipopolysaccharide and peptidoglycan activate Kupffer cells through Toll like receptor 4, which leads to release of tumor necrosis factor alpha and interleukin 6 and to activation of hepatic stellate cells. Over time, these responses drive fibrogenesis (Seki et al., 2011; Mak and Shekhar, 2025). A review of spontaneous bacterial peritonitis highlights that impaired permeability allows migration of bacteria and release of pathogen associated molecular patterns that stimulate macrophages (Rooney et al., 2024; Murayama et al., 2025). Thus, failure of the gut barrier is central in cirrhosis and it sustains bacterial invasion and systemic inflammation.

### 2.3 Inflammatory and fibrogenic loops

The influx of microbe-derived products into the liver sustains inflammation and promotes fibrosis in cirrhosis (Simbrunner et al., 2023; Costa et al., 2025a). Endotoxin activates Kupffer cells—inducing tumor necrosis factor-α, interleukin-6, and reactive oxygen species—which injure hepatocytes and activate hepatic stellate cells (Nakadate et al., 2025; Zhao et al., 2025). Lipopolysaccharide signals via Toll-like receptor 4 (TLR4) and acts as a potent fibrogenic stimulus; mice lacking TLR4 are relatively protected from fibrosis progression. In addition, peptidoglycans and bacterial DNA prime inflammasomes, amplifying chronic sterile inflammation (Blevins et al., 2022). Together, these events establish a vicious gut–liver cycle in which dysbiosis and barrier failure promote translocation, hepatic immune activation, and fibrogenesis. Reciprocal effects of worsening liver function on the gut milieu—including bile acid perturbation and consequences of portal hypertension—are addressed in Section 4.2.3, which focuses on hemodynamics and clinical sequelae (Bharti et al., 2024; Lombardi et al., 2024). Breaking this loop is a central therapeutic goal.

## 3 Microbial metabolites in cirrhosis

Gut microbes generate diverse metabolites that modulate host physiology. In cirrhosis, the balance of these metabolites shifts toward pro inflammatory and hepatotoxic profiles. Key classes include short chain fatty acids, abbreviated as SCFAs, bile acids, tryptophan derived metabolites such as indoles, serotonin, and kynurenine, as well as ammonia and ethanol.

### 3.1 Short-chain fatty acids

Short chain fatty acids, namely acetate, propionate, and butyrate, are the primary products of bacterial fermentation of dietary fiber in the gut (Liu et al., 2023). They maintain intestinal health by fueling colonocytes, strengthening tight junctions, and modulating mucosal immunity (Peng et al., 2009). Multiple studies from 2022 to 2024 show that fecal and serum concentrations of short chain fatty acids are significantly reduced in cirrhosis (Nie et al., 2023). For example, Tarasenko and colleagues reported that total fecal short chain fatty acids and each of acetate, propionate, and butyrate were markedly lower in patients with cirrhosis than in healthy controls, with P less than 0.001 (Cao et al., 2023). The depletion was more pronounced in cirrhosis of metabolic origin than in alcohol associated cirrhosis. Another cohort reported diminished fecal butyrate and propionate that correlated with disease severity (Wang et al., 2023). Reduced levels of short chain fatty acids impair barrier integrity and foster inflammation. Consistent with this, patients with cirrhosis show loss of Lachnospiraceae and Ruminococcaceae, which are the principal producers of butyrate in the colon. The consequences are multifold. The epithelium becomes weaker with higher permeability, and signaling mediated by short chain fatty acids that restrains inflammation is diminished. Clinically, lower levels of short chain fatty acids are linked to worse liver function, higher endotoxemia, and more inflammatory profiles (Rodrigues et al., 2024). Therefore, deficiency of short chain fatty acids in cirrhosis both reflects ongoing disease processes and contributes to their progression. This conclusion synthesizes evidence from human cross sectional and prospective cohort studies. These studies integrate stool and serum based metabolomics, both targeted and untargeted, with microbiome profiling using 16S rRNA gene sequencing or shotgun metagenomics. The findings correlate with clinical severity and are supported by mechanistic and animal data on epithelial barrier and immune pathways.

### 3.2 Bile acids

Bile acids, abbreviated as BA, are cholesterol derived amphipathic molecules secreted by the liver and extensively modified by gut bacteria (Hsu and Schnabl, 2023; Fleishman and Kumar, 2024). They signal through farnesoid X receptor and Takeda G protein coupled receptor 5 to regulate lipid metabolism, glucose homeostasis, and intestinal integrity (Song et al., 2025). In cirrhosis, bile acid homeostasis is disrupted. Cholestasis and dysbiosis reduce total bile acid flow and alter bile acid composition in the gut (Hsu and Schnabl, 2023). Recent reviews report that decreased luminal bile acid concentration diminishes farnesoid X receptor stimulation in enterocytes, which weakens the epithelial barrier and permits overgrowth of pathogens (Song et al., 2025). Dysbiotic bacteria further deconjugate and aberrantly modify bile acids, generating toxic bile acid species (Fuchs et al., 2025). Clinically, patients with cirrhosis show higher serum bile acids and lower levels of beneficial secondary bile acids, which correlate with cognitive impairment and systemic inflammation (Wang et al., 2016; Buckholz and Brown, 2024). Modulation of bile acid signaling is a therapeutic target. Farnesoid X receptor agonists such as obeticholic acid aim to restore bile acid and microbiota balance, and intestinal alkaline phosphatase, an enzyme that detoxifies lipopolysaccharide, is influenced by bile acid signaling (Verbeke et al., 2014; Adorini and Trauner, 2023). This synthesis integrates evidence from human cross sectional and cohort metabolomics studies, targeted and untargeted bile acid profiling, receptor signaling assays for FXR and TGR5, and microbiome sequencing with functional metagenomics. Together with mechanistic and animal models, these approaches link epithelial barrier integrity, dysbiosis, and inflammation to clinical outcomes. In summary, dysregulation of bile acids in cirrhosis fosters dysbiosis and barrier failure, drives disease progression, and provides a rationale for therapies that target the farnesoid X receptor.

### 3.3 Tryptophan metabolites

Dietary tryptophan is metabolized by host cells into serotonin, melatonin, and kynurenine, and by gut bacteria into indoles and related derivatives (Gabela et al., 2025). Indole 3 propionic acid, abbreviated as IPA, indole 3 acetic acid, abbreviated as IAA, and related indoles are mainly produced by microbes and they help maintain mucosal health by activating the aryl hydrocarbon receptor, abbreviated as AhR (Fu et al., 2023; Li et al., 2024). In cirrhosis, indole producing bacteria are depleted and concentrations of indoles decline in serum and in the intestinal lumen (Sharma et al., 2024). For example, Ilha and colleagues reported that obese patients with liver fibrosis had lower serum IPA, and that IPA exposure in cell culture induced apoptosis and functional inactivation of hepatic stellate cells (Ilha et al., 2025a). These findings suggest that IPA is hepatoprotective and that loss of IPA may promote fibrogenesis (Zhao et al., 2019). Other studies report that cirrhosis shifts tryptophan metabolism toward the host kynurenine pathway and increases concentrations of neurotoxic metabolites (Zeng et al., 2024). Metabolites in the serotonin pathway also decline. These conclusions are supported by human cross sectional and cohort metabolomics, targeted and untargeted profiling of tryptophan and indole metabolites, microbiome sequencing with functional metagenomics, and receptor assays for AhR. Mechanistic and animal models further link these pathways to clinical outcomes. In summary, cirrhosis is associated with lower levels of beneficial indoles and serotonin and with higher levels of kynurenines, and these changes are linked to complications such as hepatic encephalopathy. The gut microbiome likely contributes. Loss of indole producing Lactobacillus and Clostridium species diminishes production of indoles in the gut, whereas urea splitting bacteria increase kynurenine production (Rai et al., 2015; Jakhar et al., 2024). These metabolic shifts may weaken gut immunity and promote neuroinflammation.

### 3.4 Ammonia

Ammonia is generated in the intestine by bacterial urease activity and by deamination of amino acids (Luo et al., 2023). Under physiological conditions, the liver converts ammonia to urea, which maintains low circulating levels (Jin et al., 2025). In cirrhosis, impaired hepatic clearance and portosystemic shunting lead to hyperammonemia, which is a key driver of hepatic encephalopathy (Trivedi et al., 2024). Emerging evidence implicates the gut microbiome in ammonia production and clearance (Jakhar et al., 2024). Urease producing bacteria such as Enterobacteriaceae, Proteus, and Streptococcus salivarius are enriched in cirrhosis and contribute to excess ammonia (Liu et al., 2025). A recent review summarized how gut dysbiosis alters ammonia metabolism. Loss of commensal taxa that can consume ammonia, such as Bifidobacteria, together with expansion of ammonia producing bacteria, increases blood ammonia (Jakhar et al., 2024). Ammonia itself impairs brain function by inducing astrocyte swelling and by disrupting neurotransmission, and it acts together with lipopolysaccharide to trigger neuroinflammation (Rama Rao et al., 2010; Aldridge et al., 2015). Clinically, interventions that lower intestinal ammonia, such as lactulose and rifaximin, improve hepatic encephalopathy, which underscores the role of the microbiome (Oriko et al., 2025). This synthesis integrates evidence from human cross sectional and cohort physiology, stool and serum ammonia measurements, urease activity assays, microbiome sequencing combined with functional metagenomics to identify urease pathways, and neurophysiology studies of astrocyte swelling and neurotransmission. Interventional trials further show clinical improvement with ammonia lowering therapies, including lactulose and rifaximin. Thus, ammonia is both a product and a mediator of disturbances along the gut liver axis in cirrhosis.

### 3.5 Endogenous ethanol

Endogenous ethanol arises from fermentation of dietary carbohydrates by gut microbes, notably Klebsiella pneumoniae, Escherichia coli, and various yeast species (Yuan et al., 2019; Xue et al., 2023). Under dysbiosis, overgrowth of high alcohol producing strains can elevate systemic ethanol and mimic low level alcohol toxicity (Gan et al., 2023). This phenomenon is well documented in metabolic liver disease without cirrhosis, and it likely contributes to the pathogenesis of cirrhosis. Bacteria such as Klebsiella species have been isolated from patients with intestinal ethanol overproduction and with metabolic dysfunction associated fatty liver disease, and they can cause spikes in blood ethanol (Cordell, 2025). Elevated ethanol increases intestinal permeability by altering tight junctions and generates oxidative stress in hepatocytes through cytochrome P450 2E1, which exacerbates injury (Yan et al., 2023). Although data in cirrhosis are limited, endogenous ethanol production is plausibly higher in dysbiotic patients with cirrhosis and may aggravate hepatic inflammation and steatosis, particularly in metabolic cirrhosis (Pasta et al., 2025). This synthesis integrates human cross sectional and cohort data, quantitative blood ethanol measurements, isolation and characterization of alcohol producing microbes, microbiome sequencing with functional metagenomics, and cell and animal studies of epithelial permeability and hepatocellular oxidative stress, which together support the conclusion below. At minimum, ethanol production by gut pathogens serves as a marker of severe dysbiosis.

## 4 Clinical correlations and complications of cirrhosis

### 4.1 Microbiome and metabolite biomarkers

The gut microbiome and metabolome provide candidate biomarkers for the diagnosis, staging, and prognosis of cirrhosis (Acharya and Bajaj, 2019; Tang et al., 2022). Cross sectional studies have identified microbial signatures that separate patients with cirrhosis from healthy individuals and from earlier stages of liver disease (Oikonomou et al., 2018). For example, one study reported higher fecal levels of Enterobacteriaceae and Veillonellaceae and lower levels of Lachnospiraceae in patients with cirrhosis compared with healthy controls (Acharya and Bajaj, 2019; Liu et al., 2022). Machine learning models can use microbiome profiles to predict the etiology of cirrhosis, distinguishing metabolic dysfunction associated fatty liver disease from alcohol related disease (Liu et al., 2022). Metabolomic screening likewise shows distinct patterns in cirrhosis. Levels of short chain fatty acids are reduced, bile acid ratios are altered, and aromatic amino acid metabolites and ammonia are elevated in stool and in blood, and these changes correlate with disease severity (Pelle et al., 2022; Xu et al., 2023). Several groups have proposed a cirrhosis dysbiosis ratio that tracks with the Model for End Stage Liver Disease score and with the Child Pugh score (Bajaj et al., 2014). Moreover, specific microbial signatures may predict complications. High abundance of Streptococcus salivarius correlates with hyperammonemia and with the risk of hepatic encephalopathy (Won et al., 2022). Although still experimental, combining microbiome profiles with serum metabolites may improve noninvasive assessment of cirrhosis and of decompensation, and may complement fibrosis based scores (Acharya and Bajaj, 2021). Future work aims to determine which bacterial taxa or metabolites most reliably signal progression to decompensation or an increased risk of hepatocellular carcinoma (**Table 2**).

**Table 2:** Diagnostic and prognostic biomarkers related to the gut–liver axis in cirrhosis.

Biomarker/signature	Platform	Endpoint	Performance (typical)	Notes/limitations
Stool dysbiosis index (16S)	16S rRNA sequencing	Cirrhosis vs controls; decompensation risk	AUC commonly 0.70–0.90 (varies)	Needs etiology/medication adjustment
Oralization signature (Streptococcus/Veillonella ↑)	Shotgun metagenomics or 16S	Portal hypertension, HE	Signal replicates across cohorts	Duodenal aspirate/stool concordance varies
Bile acid ratios (DCA : CA; conjugated/unconjugated)	Targeted metabolomics	Decompensation/HE	Good discrimination in several studies	Assay standardization needed
SCFA panel (fecal butyrate/propionate)	Targeted metabolomics	Barrier integrity; HE	Lower in cases	Diet strongly confounds
Plasma LBP / EndoCAb	Immunoassays	Endotoxemia; infection risk	Useful adjunct markers	Not microbiome-specific
Composite multi-omics scores	Metagenome and metabolome	Prognosis; variceal risk	High accuracy in pilot reports	External validation required

### 4.2 Microbiota links to cirrhosis complications

#### 4.2.1 Hepatic encephalopathy

Hepatic encephalopathy is closely linked to gut dysbiosis (Rai et al., 2015; Trebicka et al., 2021). Hyperammonemia driven by gut bacteria is a classic driver of hepatic encephalopathy, and dysbiosis often increases the intestinal ammonia load (Jakhar et al., 2024). Recent studies identify specific pathobionts in hepatic encephalopathy. For example, overgrowth of Streptococcus salivarius is associated with urease driven ammonia production in patients with minimal hepatic encephalopathy (Won et al., 2022; Nomura et al., 2025). Endotoxemia arising from Gram negative overgrowth also contributes to neuroinflammation (Kalyan et al., 2022). In minimal hepatic encephalopathy, patients with cirrhosis show higher fecal Veillonella and Enterobacteriaceae, reduced Ruminococcaceae, and increased lipopolysaccharide. Therapies that restore eubiosis ameliorate hepatic encephalopathy. Rifaximin and lactulose reduce ammonia producing taxa, and probiotics increase Lactobacillus and Bifidobacterium (Bloom et al., 2021). Clinical trials show that microbiome modulating treatments such as lactulose, rifaximin, probiotics, synbiotics, and fecal microbiota transplantation improve cognition and reduce recurrence of hepatic encephalopathy (Zacharias et al., 2023a; Oriko et al., 2025). A recent randomized trial of fecal microbiota transplantation for recurrent hepatic encephalopathy reported an acceptable safety profile and fewer episodes than placebo, based on a 2025 abstract. Overall, hepatic encephalopathy exemplifies the gut liver brain axis in cirrhosis, in which gut derived toxins such as ammonia and gamma aminobutyric acid analogs together with inflammation cause neuropsychiatric effects.

#### 4.2.2 Spontaneous bacterial peritonitis

Spontaneous bacterial peritonitis arises when gut bacteria translocate into ascitic fluid (U et al., 2024). Dysbiosis and a leaky intestinal barrier in cirrhosis directly predispose to spontaneous bacterial peritonitis (Maccauro et al., 2023). Observational studies show that patients with cirrhosis and spontaneous bacterial peritonitis often harbor overgrowth of Enterobacteriaceae and Enterococcus in the gut, mirroring organisms isolated from infected ascites (Khoury et al., 2020). Impairment of the intestinal barrier allows bacteria and their extracellular vesicles to reach the peritoneal cavity (Khoury et al., 2020). Higher serum lipopolysaccharide and detectable bacterial DNA are associated with episodes of spontaneous bacterial peritonitis (González-Navajas et al., 2008). In longitudinal cohorts, patients with recurrent spontaneous bacterial peritonitis tend to show more severe dysbiosis and greater intestinal permeability. Thus, gut microbiota composition may help predict risk. For example, enrichment of Klebsiella and Clostridium species in stool has been linked to infections (Furey et al., 2023). No large contemporary randomized trial of probiotics for prevention of spontaneous bacterial peritonitis has shown conclusive benefit, although small studies suggest that probiotics can reduce markers of bacterial translocation (Macnaughtan et al., 2020). Overall, spontaneous bacterial peritonitis reflects failure of the gut barrier in cirrhosis.

#### 4.2.3 Portal hypertension and other complications

Mechanistic details of microbial translocation–driven immune activation and fibrogenesis are summarized in Section 2.3; here we focus on hemodynamic consequences and clinical sequelae. Systemic inflammation driven by gut dysbiosis contributes to the development and maintenance of portal hypertension and its sequelae, including ascites, variceal bleeding, and hepatorenal syndrome (Costa et al., 2025a). In cirrhosis, elevated circulating lipopolysaccharide and tumor necrosis factor-α promote splanchnic vasodilation and exacerbate portosystemic shunting (Iwakiri and Trebicka, 2021). Reduced short-chain fatty acids may impair vascular function (Brosolo et al., 2024). Microbiome-targeted strategies are being explored to lower portal pressure, including rifaximin as an adjunct to non-selective β-adrenergic blockade (Lim et al., 2017). In parallel, activation of hepatic stellate cells via lipopolysaccharide–Toll-like receptor 4 (TLR4) signaling accelerates fibrosis, further increasing portal pressure (Pradere et al., 2010). Thus, although more indirect than hepatic encephalopathy or spontaneous bacterial peritonitis, the gut–liver axis can amplify portal hypertension. Altered gut microbiota has also been associated with hepatic hydrothorax and renal dysfunction in cirrhosis, plausibly through spillover of microbial products and systemic inflammation (Khemichian et al., 2025). Microbiome- and metabolite-based signals linked to portal hypertension, alongside the detection matrix and evidence level, are summarized in **Table 3**.

**Table 3:** Complication-linked microbiome cues.

Complication	Taxon/Metabolite	Detection matrix	Level of evidence
Hepatic encephalopathy (HE)	Enterobacteriaceae	Stool / oral	Cohort / cross-sectional; decreases with HE therapy
HE	Streptococcus	Stool / oral	Cohort / cross-sectional
HE	Veillonella	Stool / oral	Cohort / cross-sectional
HE	Ruminococcaceae, Lachnospiraceae	Stool	Cohort / cross-sectional
HE	Short-chain fatty acids (butyrate, propionate)	Stool / serum	Cohort / cross-sectional; mechanistic support
HE	Ammonia	Plasma / serum	Physiologic/clinical; RCT-supported reduction with lactulose/rifaximin
Spontaneous bacterial peritonitis (SBP)	Enterobacteriaceae	Ascites / stool	Clinical microbiology / cohort
SBP	Enterococcus	Ascites	Clinical microbiology / cohort
Portal hypertension	Streptococcus	Stool / oral	Cross-sectional / cohort; mechanistic support
Portal hypertension	Veillonella	Stool / oral	Cross-sectional / cohort; mechanistic support
Portal hypertension	Short-chain fatty acids	Stool	Cross-sectional / cohort; mechanistic support

### 4.3 Comparative evaluation of gut-targeted interventions

To align mechanisms with clinical decision-making, we compare core gut-targeted interventions by mechanism, typical clinical effects, evidence strength, safety, and best-use scenarios. We also indicate when combination therapy is favored (**Table 4**).

**Table 4:** Comparative summary of gut-targeted interventions in cirrhosis.

Intervention	Primary mechanisms	Typical clinical effects	Evidence type and strength	Safety and tolerability
Probiotics	Rebalance community, decrease urease-positive pathobionts, increase SCFAs, modulate immune tone	Prevention or reduction of minimal or overt HE in selected settings; improvement in dysbiosis biomarkers	Mixed randomized and observational human data; heterogeneity across strains and dosing	Generally well tolerated; rare bacteremia in high-risk hosts; quality control varies
Lactulose	Colonic acidification, ammonium trapping, catharsis	Treatment and secondary prevention of HE; improves neurocognitive endpoints	Multiple randomized trials and guidelines support use	GI adverse effects, dehydration risk; adherence limits
Rifaximin	Gut-selective antimicrobial, reduces urease producers and toxic metabolites, stabilizes microbiome function	Reduces HE episodes and hospitalization when added to lactulose	Strong randomized and extension data for recurrent HE	Well tolerated; cost and stewardship considerations
FMT	Restore community structure and function; enhance colonization resistance	Signals for HE improvement and fewer hospitalizations in selected cohorts	Early randomized and real-world studies; protocols vary	Requires rigorous donor screening; infection-transmission safeguards essential
Bile-acid pathway modulators, including FXR agonists	Normalize BA signaling, strengthen epithelial barrier, reduce inflammation, rebalance BA–microbiome axis	Improvements in biomarkers and disease activity indices; symptom changes context-dependent	Early- to mid-phase interventional studies	Pruritus, lipid effects; monitoring needed

Lactulose remains the backbone for treatment and prevention of hepatic encephalopathy. It acidifies the colon, traps ammonia as ammonium, and accelerates transit (Bloom and Tapper, 2023a). However, it does not lower portal pressure (Montagnese et al., 2022). Rifaximin is a gut-selective, minimally absorbed antibiotic that provides consistent incremental benefit as an add-on to lactulose by reducing ammonia-producing bacteria and neuroinflammatory metabolites (Bass et al., 2010). Cost and antimicrobial stewardship remain important considerations (Hoilat et al., 2022). Probiotics, prebiotics, and synbiotics can modestly stabilize the microbiome and reduce dysbiosis markers. Effects are strain and dose dependent and often attenuate in advanced decompensation (Wibawa et al., 2023). Use with caution in severe immunosuppression. Fecal microbiota transplantation may restore community structure and reduce recurrent encephalopathy and hospitalizations in carefully selected patients. It requires rigorous donor screening and specialized oversight. Bile acid pathway modulators, such as FXR agonists, strengthen epithelial integrity, reduce inflammation, and normalize interactions between the microbiota and bile acids. Early to mid-phase signals are encouraging, but pruritus and lipid changes require monitoring (Almeqdadi and Gordon, 2024).

Evidence is strongest for lactulose and rifaximin based on randomized and controlled trials. It is mixed for probiotics and synbiotics and early for fecal microbiota transplantation and bile acid modulators (Bass et al., 2010; Wibawa et al., 2023). A pragmatic approach is stepwise and phenotype directed. Use lactulose plus rifaximin for recurrent encephalopathy. Add probiotic or synbiotic support selectively. Reserve fecal microbiota transplantation for recurrence despite standard care in experienced centers. Layer bile acid modulators when cholestasis or bile acid dysregulation predominates (Zacharias et al., 2023a).

## 5 Microbiome-targeted therapies and interventions

**Figure 2:**
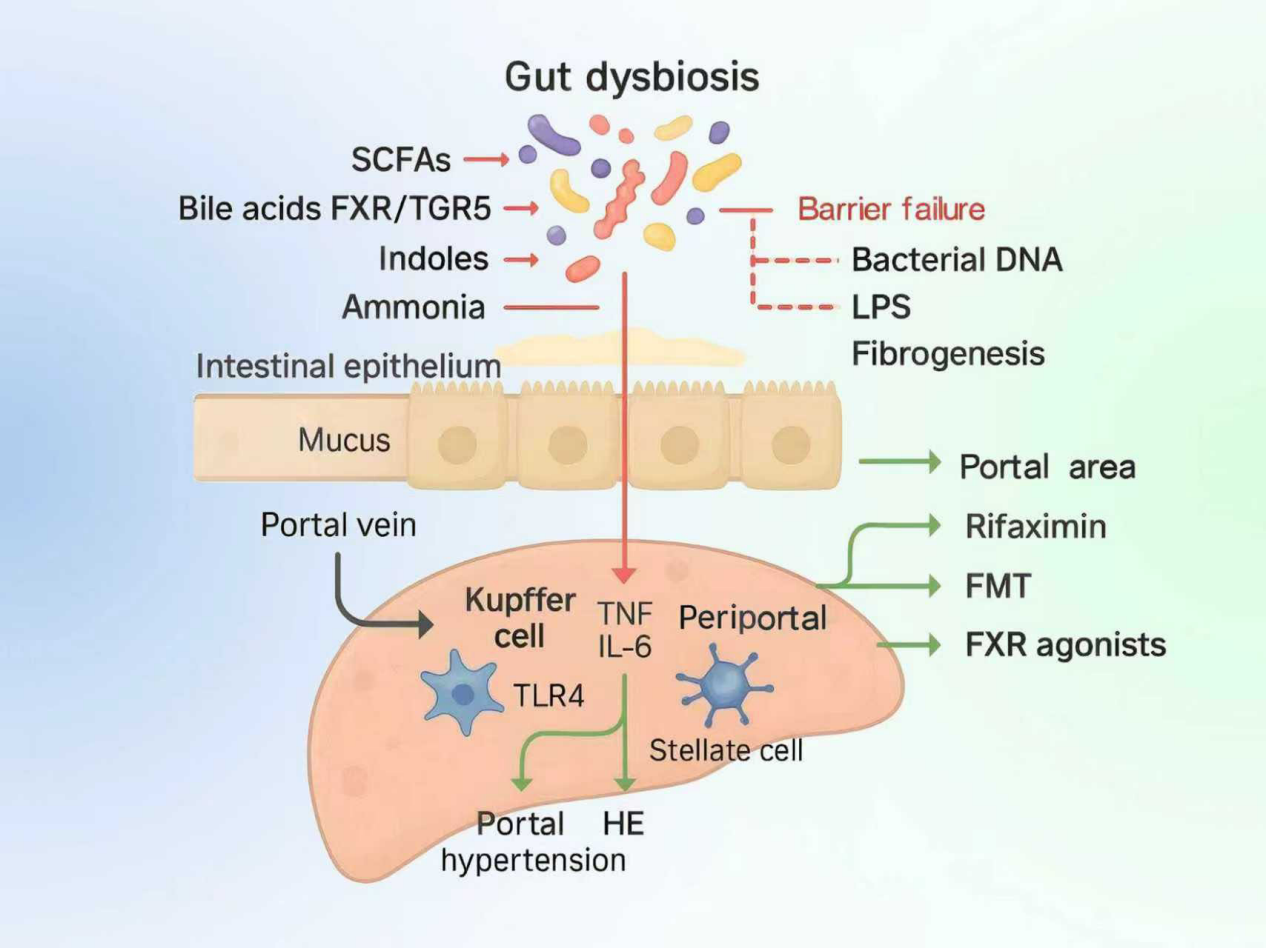
Dysbiosis–inflammation cascade and therapeutic targets. Gut dysbiosis lowers SCFAs, perturbs bile-acid/indole signaling, raises ammonia, and weakens the epithelial barrier, allowing bacterial DNA/LPS to translocate via the portal vein. In the liver, TLR4-mediated Kupffer-cell activation elevates TNF/IL-6 and activates stellate cells, driving fibrosis, portal hypertension, and hepatic encephalopathy. Rifaximin/FMT act upstream to rebalance the microbiota and reduce endotoxin load, while FXR agonists restore bile-acid signaling in periportal/portal regions.

We map therapeutic leverage points to the dysbiosis–inflammation cascade (**Figure 2**), linking rifaximin, FMT and FXR agonists to defined nodes.

### 5.1 Probiotics, prebiotics and synbiotics

Probiotics, defined as live beneficial bacteria, and prebiotics, defined as nondigestible fibers, have been extensively studied (Rocco et al., 2021). Commonly studied probiotics include Lactobacillus, Bifidobacterium, Clostridium butyricum, and Saccharomyces boulardii. Meta analyses and clinical trials indicate that probiotics can modestly improve liver biochemical tests and reduce episodes of hepatic encephalopathy (Zhou et al., 2024). For example, a multicenter randomized controlled trial reported that Lactobacillus GG reduced recurrence of hepatic encephalopathy and decreased endotoxemia, and smaller trials suggested that combined probiotic and fermenter blends improved minimal hepatic encephalopathy (Xia et al., 2018). Probiotics appear to lower ammonia and tumor necrosis factor alpha, likely by displacing urease producing bacteria and by enhancing intestinal barrier integrity (Bloom et al., 2021). Prebiotics such as lactulose and inulin provide additional benefit by acidifying the intestinal lumen, which traps ammonia, and by supplying substrates for commensal bacteria (Bloom and Tapper, 2023a). Trials of synbiotic combinations show additive effects on cognition and on markers of portal pressure. A recent cohort from China reported that patients with cirrhosis who received a multispecies probiotic containing Bifidobacterium, Lactobacillus, and Enterococcus for one month showed improved albumin and bilirubin compared with controls (Xu et al., 2024). Nonetheless, the effects of probiotics are generally modest and depend on the specific strain. Safety in cirrhosis is generally acceptable, although caution is warranted in decompensated patients because bacteremia can occur, albeit rarely (Kullar et al., 2023).

### 5.2 Non-absorbable antibiotics

Gut specific antibiotics have long been part of standard care for cirrhosis. Lactulose, a nonabsorbable disaccharide, remains first line therapy for hepatic encephalopathy. It is fermented by colonic bacteria to acidify the intestinal lumen and to trap ammonia, and it shifts the gut microbiota toward Bifidobacterium and Lactobacillus (Moratalla et al., 2017). Recent studies show that lactulose reduces translocation of bacterial DNA and decreases systemic cytokines. Rifaximin is a nonabsorbable rifamycin antibiotic that is approved to prevent recurrence of hepatic encephalopathy (Bass et al., 2010). It broadly suppresses enteric pathogens while sparing many commensal organisms (Koo and DuPont, 2010). Contemporary trials indicate that rifaximin lowers endotoxin levels and reduces ammonia production without clear signals of clinically meaningful antimicrobial resistance (Kaji et al., 2017). Mechanistically, rifaximin upregulates tight junction proteins and downregulates bacterial virulence genes, which helps restore epithelial barrier function (Jin et al., 2018). Long term use of rifaximin has been associated with fewer hospitalizations for hepatic encephalopathy in observational studies and may also slow progression of fibrosis, possibly through reduced inflammation (Oey et al., 2019). Combination therapy with rifaximin and lactulose is superior to lactulose alone for preventing hepatic encephalopathy. One caveat is cost and the potential for dysbiosis with broader spectrum antibiotic use. Even so, the gut specific action of rifaximin and its minimal systemic absorption make it relatively safe.

### 5.3 Fecal microbiota transplantation (FMT) and engineered bacteria

Fecal microbiota transplantation, abbreviated as FMT, transfers processed stool from a healthy donor and has emerged as a transformative approach to reset the microbiome. Preliminary trials in cirrhosis, mostly in patients with hepatic encephalopathy, have yielded encouraging results (Bajaj et al., 2017). A landmark study in 2018 reported that patients with cirrhosis who received oral FMT after antibiotic preparation had improved cognitive scores and lower recurrence of hepatic encephalopathy at five months compared with placebo (Bajaj et al., 2019). Larger randomized trials are ongoing (Shawcross and Patel, 2025). FMT may restore microbial diversity, increase producers of short chain fatty acids, and reduce taxa that generate ammonia. A recent open label study in alcohol associated cirrhosis found that FMT was safe and provided benefit, including improvements in the Model for End Stage Liver Disease score and in inflammatory markers (Sharma et al., 2022). Genetically engineered probiotics are also under investigation, including strains designed to secrete bile acid modulators or to degrade ammonia (Sadhu et al., 2025). However, FMT in cirrhosis requires caution because immunocompromised patients may face an increased risk of infection. Overall, FMT remains promising yet experimental, and no large phase three trials have led to regulatory approval for cirrhosis. It exemplifies a broader strategy of microbiome reprogramming (Bajaj et al., 2022).

### 5.4 Bile acid pathway modulators

Given the central role of dysregulated bile acid signaling, drugs that target bile acid receptors are under active investigation. Obeticholic acid, a farnesoid X receptor agonist, is approved for primary biliary cholangitis and is being tested in cirrhosis due to metabolic dysfunction associated steatohepatitis (Fiorucci et al., 2025). In cirrhosis, activation of the farnesoid X receptor can upregulate fibroblast growth factor 19, suppress hepatic bile acid synthesis, and strengthen epithelial barrier function (Sonne, 2021). Early studies show that farnesoid X receptor agonists can reduce portal pressure and inflammation in experimental models of cirrhosis (Verbeke et al., 2014). Another approach uses inhibitors of the ileal bile acid transporter to reduce reabsorption and to reshape the bile acid pool. These agents may also shift the gut microbiome toward a healthier profile. Randomized trials of farnesoid X receptor agonists in cirrhosis, including trials for prevention of variceal bleeding, are ongoing, although safety concerns such as dyslipidemia and pruritus persist (Fiorucci et al., 2025). In addition, ursodeoxycholic acid and tauroursodeoxycholic acid can modulate the intestinal microbiota, and ursodeoxycholic acid has reversed certain microbiota changes in primary biliary cholangitis (Mao et al., 2024). In summary, therapies that modulate bile acid signaling represent an active frontier in gut liver therapeutics.

### 5.5 Emerging directions

Recent advances have expanded microbiome therapeutics beyond conventional probiotics and antibiotics, introducing engineered probiotics, metabolite-based interventions, and CRISPR-guided microbiome editing (Brödel et al., 2024). Engineered probiotics constitute a new generation of living biotherapeutics. Synthetic biology allows the programming of probiotic strains such as Escherichia coli Nissle 1917 and Lactobacillus reuteri to perform targeted functions including ammonia detoxification, bile acid modulation, and anti-inflammatory cytokine secretion (Kurtz et al., 2019). These engineered strains actively restore microbial balance and hepatic homeostasis, providing disease-specific and programmable therapeutic effects (Zou et al., 2023).

Targeted metabolite supplementation aims to replace or mimic essential metabolites that are depleted in cirrhosis. Supplementation with short-chain fatty acids such as sodium butyrate enhances epithelial barrier integrity and reduces systemic inflammation (Fogacci et al., 2024), while indole-3-propionic acid and its derivatives activate AhR signaling to suppress fibrogenesis and protect hepatocytes (Ilha et al., 2025a). Recent preclinical studies combining butyrate and indole-3-propionic acid analogs demonstrate synergistic improvement in intestinal defense and inhibition of TLR4-mediated inflammation, supporting metabolite therapy as a precise and safe adjunct to microbiome modulation (Xu et al., 2025a).

CRISPR-based microbiome editing represents an emerging frontier that enables *in situ* manipulation of gut microbial communities at the gene level (Lam et al., 2021). Phage or plasmid-delivered CRISPR systems can selectively silence virulence and antibiotic resistance genes in pathogenic bacteria such as Enterobacteriaceae while preserving beneficial taxa (Nath et al., 2022). Commensal strains can also be engineered to secrete therapeutic metabolites or to express biosensors that respond dynamically to intestinal environmental cues. Although still in preclinical stages, CRISPR-guided precision microbiome surgery exemplifies the transition from passive correction to active molecular-level ecosystem engineering.

Collectively, these innovations including engineered microbes, metabolite restoration, and genome-level editing define the next generation of microbiome therapeutics. As delivery systems, biosafety, and regulatory frameworks continue to mature, these technologies hold the potential to transform cirrhosis management from empirical modulation to rational and personalized microbiome engineering (Gencay et al., 2024).

## 6 Challenges and future directions

Although the gut liver axis offers new opportunities, several challenges still impede progress. Population diversity is one important challenge. The composition of the gut microbiome is shaped by geography, diet, ethnicity, and the etiology of liver disease. Biomarker signatures identified in one cohort often fail to replicate in other populations. For example, microbiota associated with cirrhosis in China differ from those reported in European cohorts (Huan et al., 2021). Therefore, universal microbial markers remain elusive, and personalized reference ranges may be required. Causal inference remains challenging. Most human studies are observational and primarily associative. Although animal models and fecal microbiota transplantation studies implicate the microbiome in pathogenesis, it remains difficult to prove that specific bacteria or metabolites cause changes in cirrhosis rather than merely reflect them. Advanced models such as humanized gnotobiotic mice and organoids may help to dissect mechanisms.

The safety of microbiome based therapies is another major concern. Antibiotics such as rifaximin can alter the intestinal resistome, and the long term effects on commensal communities remain unclear. Probiotics are generally safe, yet rare infections have been reported in immunosuppressed patients (Radcliffe et al., 2025). Fecal microbiota transplantation carries a risk of transferring pathogens or undesirable microbial genes. Regulatory frameworks for these therapies are still evolving.

**Figure 3:**
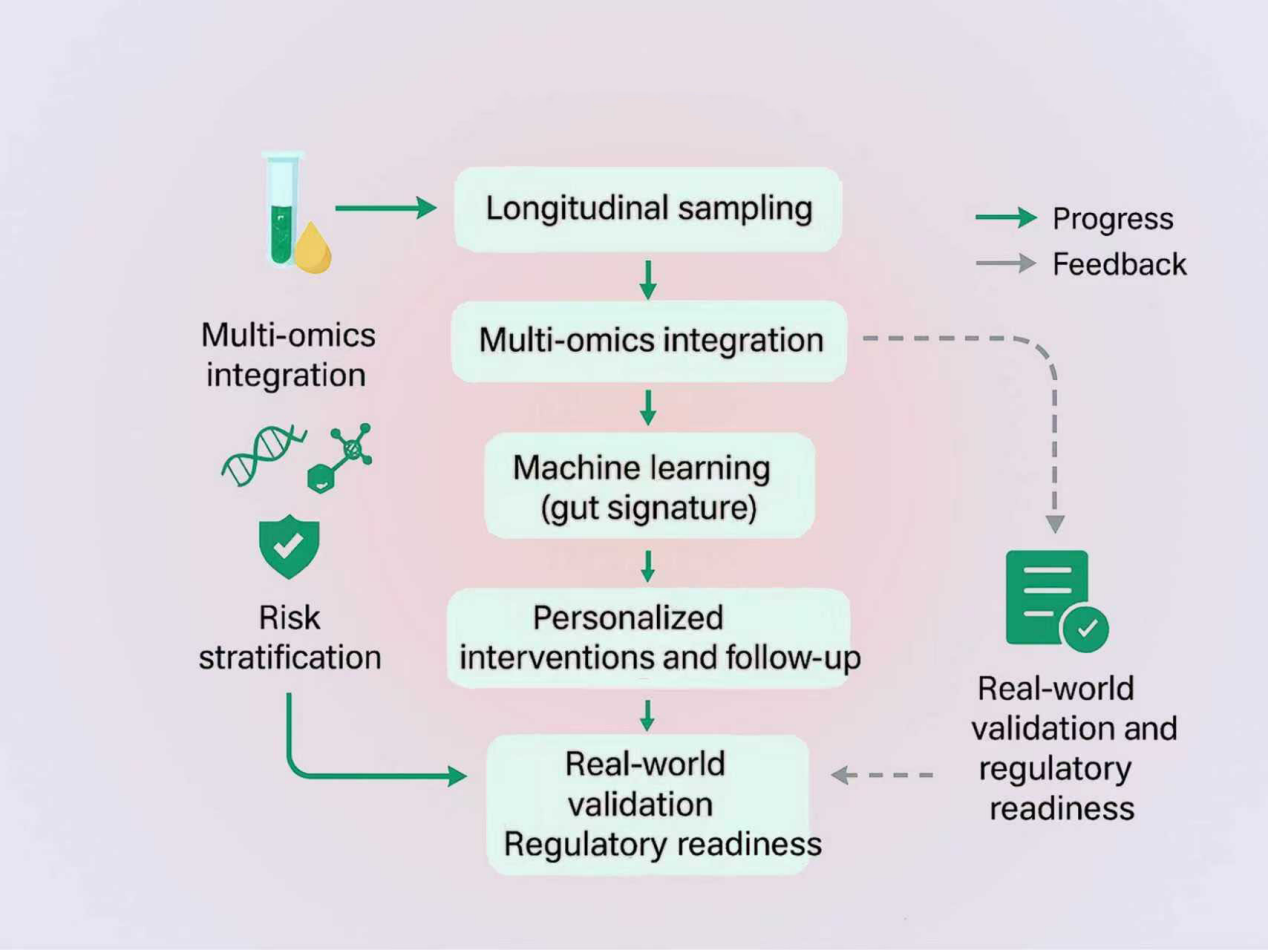
Roadmap for microbiome-informed precision care. Longitudinal sampling feeds multi-omics integration to generate an analyte-level view of the gut–host interface. Outputs inform personalized interventions and follow-up. Downstream, real-world validation and regulatory review evaluate performance, generalizability, and safety. Green solid arrows denote the forward process flow, and gray dashed arrows indicate feedback loops that return clinical evidence to earlier stages for model and workflow refinement.

Finally, precision medicine and multi omics approaches are likely to shape the field.A staged roadmap from longitudinal sampling to real-world validation is proposed to enable microbiome-informed precision care (**Figure 3**). Integrating metagenomics, metabolomics, transcriptomics, and host genomics could yield composite biomarkers and individualized therapies. Machine learning applied to microbiome datasets shows promise for early detection of cirrhosis and may guide tailored probiotics or dietary interventions. Multi omics approaches can also reveal novel interactions between microbes and the host, for example specific gut microbes that drive fibrosis through epigenetic changes. Ultimately, an individualized gut signature may inform risk stratification and targeted intervention (Pepke et al., 2024). Such personalized gut liver medicine is on the horizon, yet it will require large and longitudinal studies for validation (Dey and Choubey, 2024).

## 7 Conclusions

In summary, a large body of research from 2022 to 2025 has deepened understanding of the role of the gut microbiome in cirrhosis. Cirrhosis is consistently associated with gut dysbiosis, including loss of short chain fatty acid producing taxa and enrichment of pathogens, together with altered microbial metabolites that promote disease. Mechanistically, these changes impair intestinal barrier function and intensify hepatic inflammation and fibrogenesis. Clinically, dysbiosis correlates with the severity of cirrhosis and with complications such as hepatic encephalopathy and spontaneous bacterial peritonitis. Encouragingly, microbiome based interventions such as probiotics, rifaximin, fecal microbiota transplantation, and farnesoid X receptor agonists show potential to restore microbial balance and to improve clinical outcomes. Nevertheless, important challenges remain in translating these findings into clinical practice. Future research should define causality, identify robust microbial biomarkers across diverse populations, and optimize safe therapeutic modulation of the gut liver axis. As multi omics and precision approaches advance, targeting the gut microbiome holds promise for improving prevention, diagnosis, and treatment of cirrhosis and its complications.The integration of multi-omics and artificial intelligence-assisted analysis may ultimately propel microbiome-based stratified treatment strategies from the laboratory into clinical practice, revolutionising the management of liver cirrhosis.
